# The value of micro-ultrasound for prostate cancer screening: A retrospective real-world feasibility study

**DOI:** 10.1038/s41391-026-01075-x

**Published:** 2026-01-26

**Authors:** Johann Jakob Wendler, Jonas Bechstein, John Buckendahl, Stephan Kruck, Christian Samtleben, Bernd Uwe Liehr, Markus Porsch, Hannes Cash

**Affiliations:** 1 Clinic of Urology, Urooncology and Robotic and Focal Therapy, Medical Faculty of University Magdeburg and University Hospital Magdeburg A. ö. R. (University Medicine Magdeburg, UMM), Magdeburg, Germany; 2 Urologicum Magdeburg, Magdeburg, Germany; 3 PROURO Berlin, Berlin, Germany; 4 Department of Urology, Siloah St. Trudpert Clinic, Pforzheim, Germany

**Keywords:** Outcomes research, Cancer screening

## Abstract

**Introduction:**

Prostate cancer (PCa) screening is increasingly guided by imaging. High-resolution 29 MHz Micro-Ultrasound (MUS) offers a promising alternative to magnetic resonance imaging (MRI).

**Methods:**

We retrospectively analyzed 682 consecutive men undergoing MUS and PSA testing during routine examination. Biopsy and MRI were performed according to guideline recommendations. PSA density (PSAD)-modified negative MUS included PRI-MUS categories 1, 2, or 3 with PSAD < 0.15 ng/mL^2^; PSAD-modified positive MUS included PRI-MUS categories 3 with PSAD ≥ 0.15 ng/mL^2^, 4 or 5.

**Results:**

Median age was 59 years; median PSA 1.2 ng/mL (IQR: 0.6–3.5). Biopsies were performed in 62 men, detecting PCa in 29 (47%), including 18 (29%) clinically significant PCa (csPCa). 88 men (13%) had PSAD-modified positive MUS, yielding 15 csPCa and 7 non-clinically significant PCa (ncsPCa). Among 594 men (87%) with PSAD-modified negative MUS, 3 csPCa and 4 ncsPCa were detected. Compared to PSA-based biopsy indication ≥3 ng/mL, PSAD-modified negative MUS would have avoided 13 negative, missing two csPCa and four ncsPCa.

Compared to the MRI-based biopsy indication (PI-RADS ≥ 3, *n* = 38), PSAD-modified negative MUS (*n* = 594) would have spared 3 negative biopsies, as well as 17 (24.7% of 69) MRIs due to negative biopsy, while missing 0 cases of csPCa.

Additionally, MRI could have been omitted in 1csPCa case and 9 ncsPCa cases with positive MUS, and in 13 csPCa and 7 ncsPCa cases based on PSAD-modified positive MUS.

The PSAD-modified-PRI-MUS-based screening pathway showed a 6.29-fold (OR = 0.16) reduction in overdiagnosis and 7.22-fold (OR = 0.14) reduction in negative biopsies/ncsPCa. MUS without PSA demonstrated an OR of 7.30 to detect csPCa. PSAD-modified-PRI-MUS score demonstrated a sensitivity of 83.3%, a specificity of 59.1%, a positive predictive value of 45.5% and a negative predictive value of 89.7% for distinguishing csPCa from benign/ncsPCa findings.

**Conclusion:**

MUS enables effective PCa risk stratification in an opportunistic screening setting supporting prospective trial development.

**Trial Registration:**

This study is part of the PROSTAMUS trial, registered in the DRKS/WHO registry.

## Introduction

The incidence of prostate carcinoma (PCa) is projected to more than double by the year 2040 **[**1]. Therefore, improving the diagnostic pathway for PCa is essential to avoid unnecessary costly and invasive advanced diagnostic investigations. Screening programs are available in some European countries, but most countries only offer opportunistic screening. After a 16-year follow-up the ERSPC trial showed that prostate specific antigen (PSA) based screening reduced PCa mortality by 20% but had a risk of over diagnosing Gleason Grade Group (GG) 1 cancer [1]. With the known pitfalls of PSA based screening, the Goteborg Prostate Cancer Screening trial was able to further reduce the number of GG1 cancers by adding the magnetic resonance imaging (MRI) pathway for men with an elevated PSA > 3 ng/mL and increase the detection of clinically significant PCa (csPCa) by 67% [2].

The PROBASE trial which included young men at age 45 has shown that adding the MRI after elevated PSA of 3 ng/mL would reduce the number of unnecessary biopsies by 20% if Prostate Imaging-Reporting and Data System (PI-RADS) 3–5 were used as cut-off or by 68% for PI-RADS 4/5 at the cost of missing 13% of csPCa cases [3].

The ReImagine trial included a bolder concept comparing MRI based screening to the PSA based concept. The trial found that 67% of csPCa were detected by MRI below the PSA threshold of 3 ng/mL [4].

The future concept of PCa screening based on imaging rather than on PSA is an intriguing one. Currently, this notion is limited by the widespread availability and reimbursement of prostate MRI in many countries.

A rising image modality is high resolution 29 MHz ultrasound (Micro-Ultrasound, MUS) of the prostate, has the potential to overcome the limitations of MRI based prostate imaging. The recently published prospective multi-center OPTIMUM trial was able to demonstrate non-inferiority of MUS targeted biopsies compared to MRI targeted biopsies in detection csPCa in men at risk of prostate cancer [5].

This exploratory analysis seeks to investigate the potential of MUS in a cohort of healthy men with opportunistic screening.

## Methods

### Study design and participants

This retrospective analysis included a consecutive cohort of 682 men who underwent transrectal MUS of the prostate as part of routine, January and December 2023 in an outpatient setting. We deliberately restricted the analysis to the one-year period of 2023. Between 2018 and 2022, MUS was primarily used in an introductory and training phase and not yet applied systematically in routine prostate cancer screening. In contrast, 2023 represented the pilot phase of standardized routine application in our outpatient setting. Therefore, this period was chosen for the retrospective study in order to ensure data homogeneity and to minimize potential bias related to the learning curve.

This retrospective analysis was designed as a real-world data study derived from routine outpatient early prostate cancer screening. The study did not follow a randomized design, and not all patients underwent multiparametric MRI or prostate biopsy, as these procedures were only performed when clinically indicated. Consequently, the study cohort reflects everyday clinical practice rather than a pre-selected clinical trial population.

Patients who did not undergo biopsy typically presented with low PSA levels, negative MUS findings, and low overall clinical suspicion for prostate cancer. Patients who did not undergo MRI either had reassuring MUS/PSA results, contraindications to MRI, or declined further diagnostic work-up.

**Fig. 1:**
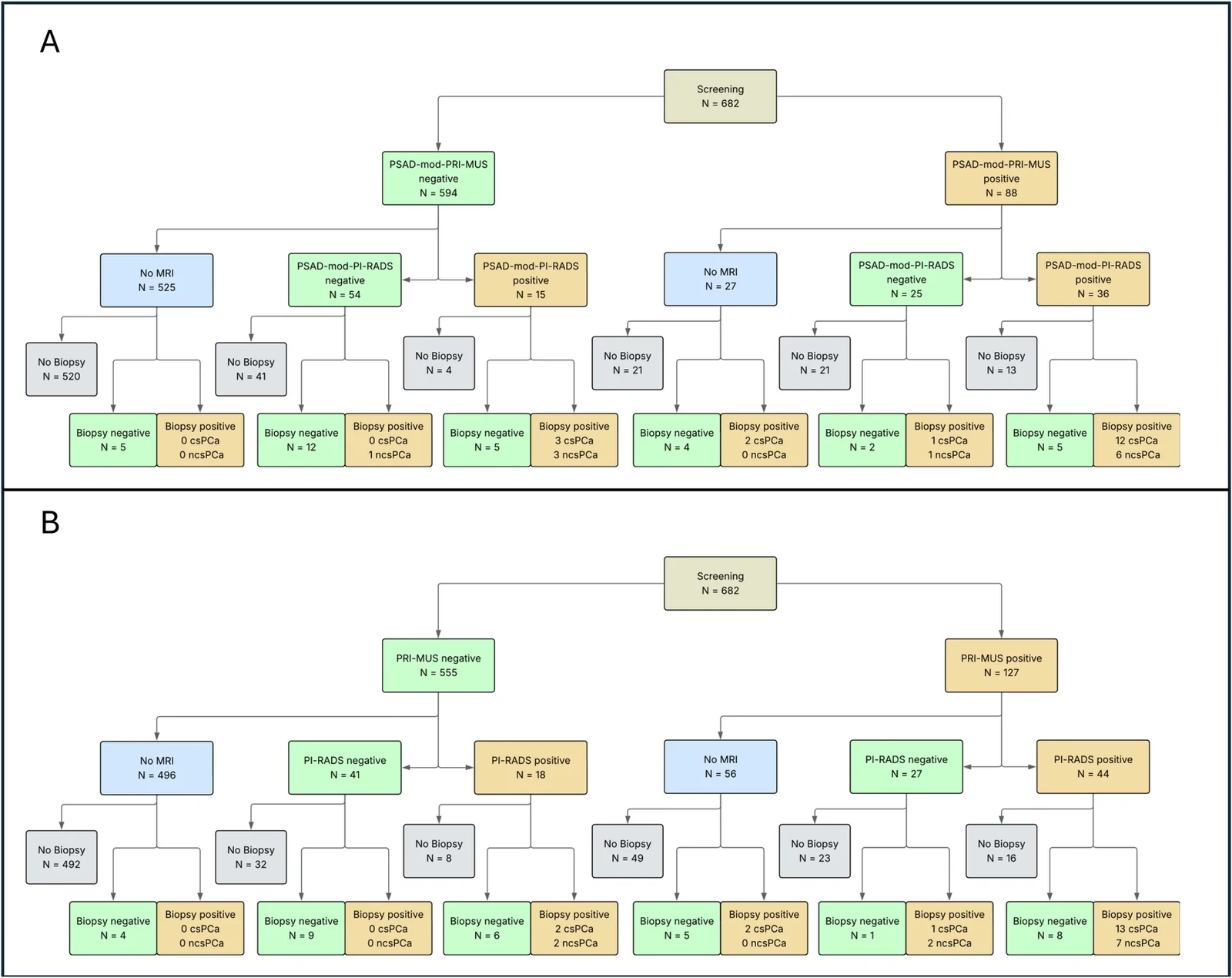
Micro-ultrasound screening pathways. **A** Screening pathway for PSAD-modified-PRI-MUS and PSAD-modified-PI-RADS. PSAD-modified negative MUS was defined as PRI-MUS categories 1, 2, 3 combined with a PSAD 0.15 ng/mL^2^. PSAD-modified positive MUS was defined as PRI-MUS categories 3 with a PSAD ≥0.15 ng/mL^2^, 4 or 5. Further imaging with MRI and where performed biopsy results are shown. **B** Screening pathway for PRI-MUS ≥ 3 and PI-RADS ≥ 3 without PSA density-modification for PRI-MUS 3 cases. Further imaging with MRI and where performed biopsy results are shown. csPCa defined as GG ≥2.

Patients were retrospectively analyzed using both PSAD-modified and non-modified diagnostic pathways. The PSAD-modified classification is depicted in Figure 1A, while the pathway without PSAD-modification is presented in Fig. 1B.

The study was approved by the institutional ethics committee, and all participants provided written informed consent. The PROSTAMUS study is registered [6] in the German Clinical Trials Register (DRKS00036777).

All men participating in the prostate cancer screening program who underwent both prostate-specific antigen (PSA) testing and micro-ultrasound (MUS) were included in the study. Patients who were not eligible for transrectal examination or whose data were incomplete or non-evaluable were excluded.

### PSA testing

According to the current German S3 guideline on prostate cancer, which recommends PSA testing as the central tool for early detection after appropriate counseling, all study participants underwent PSA testing.

### Micro-ultrasound protocol

MUS was performed in each patient using the ExactVu system with an EV29L 29 MHz side-fire transducer (Exact Imaging, Markham, Canada) by two highly experienced urologists (H.C. and J.B.). Both examiners had completed formal training with Exact Imaging and had performed more than 1000 MUS examinations since 2018. The prostate was evaluated using the PRI-MUS protocol with standardized reporting [7].

The standard diagnostic work-up included the collection of medical history, PSA testing, digital rectal examination (DRE), and transrectal MUS. In a subset of patients, PSA results were not available at the time of MUS evaluation.

### MRI and biopsy procedures

Prostate MRIs and biopsies were performed according to national guideline recommendations. All MRIs were conducted as multiparametric 3-Tesla scans with standardized reporting based on PI-RADS version 2.1 [8] in two expert centers.

All biopsies were performed via the perineal approach without antibiotic prophylaxis. In patients with positive MRI findings, targeted biopsies were guided using the ExactVu FusionVu system. A 12-core systematic biopsy scheme was applied, supplemented by 2–3 targeted cores from PI-RADS/PRI-MUS lesions.

For the evaluation of PRI-MUS 3 lesions, we followed the biopsy strategy recommended for PSA density (PSAD)-modified PI-RADS 3 lesions according to the German S3 Prostate Cancer Guideline valid during the study period [9]. Biopsy data were collected according to START criteria [10].

### Definitions

PSAD was considered suspicious at a threshold of ≥0.15 ng/mL². MRI-driven biopsy indication was defined as PI-RADS categories 3, 4, or 5. PSAD-modified positive MRI was defined as PI-RADS categories 3 combined with a PSAD ≥ 0.15 ng/mL², 4, or 5. Negative MRI was defined as PI-RADS categories 1 or 2, while PSAD-modified negative MRI included PI-RADS categories 1, 2, or 3 with a PSAD < 0.15 ng/mL².

Negative MUS was defined as PRI-MUS categories 1 and 2, and positive MUS as PRI-MUS categories 3, 4, and 5. PSAD-modified negative MUS was defined as PRI-MUS categories 1, 2, or 3 combined with a PSAD < 0.15 ng/mL², while PSAD-modified positive MUS was defined as PRI-MUS categories 3 with a PSAD ≥ 0.15 ng/mL², 4 or 5. We defined csPCa as a Gleason score (GS) ≥ 3 + 4 = 7 or GG ≥ 2.

### Study objectives

This study aimed to evaluate the diagnostic role of multiparametric ultrasound (MUS) in prostate cancer screening in a real-world outpatient setting. Given the non-randomized design and incomplete MRI or biopsy verification in all patients, the objectives should be interpreted within this practical context.

- To explore the feasibility and clinical applicability of Micro-Ultrasound (MUS) in an opportunistic prostate cancer screening setting.
- To describe correlations between MUS, PSA, PSAD, MRI, and biopsy findings in a real-world cohort.
- To estimate, in an exploratory manner, the potential of PSAD-modified MUS findings to reduce unnecessary MRI or biopsy procedures.
- To generate hypotheses for the design of future prospective validation studies.

### Statistical analysis

Data analysis was performed using PSPP software (Version 2.0.0, 1998–2023). Categorical variables were presented as frequencies and percentages, and continuous variables as medians with interquartile ranges (IQR). Comparisons between groups were performed using the chi-square test for categorical variables and ANOVA for continuous variables. A two-sided *p*-value of <0.05 was considered statistically significant for all analyses.

## Results

682 men underwent MUS and PSA testing during routine examination. The median examination time was 2.9 min (IQR 2.8 to 3.1).

Median age was 59 years and median PSA 1.2 ng/mL (IQR: 0.6–3.5). Based on the different threshold values, 133 cases showed a PSA level ≥4 ng/mL, 167 cases had PSA ≥ 3 ng/mL, and 225 cases had PSA ≥ 2 ng/mL. A PSAD of ≥0.15 ng/mL² was observed in 73 cases (Tab.4). Overall, 457 men had a PSA below 2 ng/mL of which 444 (97.2%) were PSAD-modified-PRI-MUS negative. For PRI-MUS 1-2 and PSA ≤ 2 ng/mL this was the case in 424 men (92.8%).

Of 682 patients, 88 (12.9%) were PSAD-modified-PRI-MUS positive and 594 (87.1%) were PSAD-modified-PRI-MUS negative. We found significant differences in age, PSA level, PSAD, findings of DRE, MUS-volume, number of MRI and the distribution of PI-RADS findings between the PRI-MUS groups (Table 1). Prostate MRI was performed in 130 (19.1%) patients, PI-RADS distribution is shown in Table 1. Patients without biopsy had a median PSA of 1.1 ng/ml and predominantly negative MUS findings. Patients without MRI were not referred due to low clinical suspicion (*n* = 542).

**Table 1:** Baseline participant characteristics.

Characteristic	All	PRI-MUS positive*	PRI-MUS negative*	*p* value
Number of patients (%)	682	88 (12.9)	594 (87.1)	N/A
Age [yr], median (IQR)	59 (51.0 – 66.0)	66 (61.0 – 74.8)	57 (50.0 – 64.0)	<0.001
Pos. family history of PCa (%)	65 (9.5)	5 (5.7)	60 (10.1)	0.188
Suspicious DRE (%)	28 (4.1)	24 (27.3)	4 (0.7)	<0.001
Median prostate volume [mL], (IQR)	26 (21–40)	40 (26.8 - 60)	25 (20 - 37)	<0.001
Median PSA [ng/mL], (IQR)	1.2 (0.6 – 3.6)	6.14 (4.0 - 9.1)	1.02 (0.6 - 2.3)	<0.001
Median PSAD [ng/mL²], (IQR)	0.04 (0.03 – 0.09)	0.15 (0.09 - 0.23)	0.04 (0.02 - 0.07)	<0.001
PSA ≥ 4 ng/mL (%)	133 (19.5)	63 (71.6)	70 (11.8)	<0.001
PSA ≥ 3 ng/mL (%)	167 (24.5)	69 (78.4)	98 (16.5)	<0.001
PSA ≥ 2 ng/mL (%)	225 (33.0)	75 (85.2)	150 (25.3)	<0.001
PSAD ≥ 0.15 ng/mL² (%)	73 (10.7)	42 (47.7)	31 (5.2)	<0.001
PRI-MUS 1/2 (%)	555 (81.4)	N/A	555	N/A
PRI-MUS 3 (%)	52 (7.6)	13 (14.8)	39 (6.6)	0.007
PRI-MUS 4 (%)	44 (6.5)	44	N/A	N/A
PRI-MUS 5 (%)	31 (4.5)	31	N/A	N/A
MRI (%)	130 (19.1)	61 (69.3)	69 (11.6)	<0.001
PI-RADS 1/2 (%)	68 (52.3)	23 (37.7)	45 (65.2)	0.002
PI-RADS 3 (%)	19 (14.6)	7 (11.5)	12 (17.4)	0.563
PI-RADS 4 (%)	33 (25.4)	23 (37.7)	10 (14.5)	0.002
PI-RADS 5 (%)	10 (7.7)	8 (13.1)	2 (2.9)	0.029

*DRE* digital rectal exam, *IQR* interquartile range, *MUS* Micro-ultrasound, *mpMRT* multiparametric magnet resonance imaging, *PCa* prostate cancer, *PI-RADS* Prostate Imaging Reporting and Data System, *PRI-MUS* Prostate Risk Identification using Micro-ultrasound, *PSA* prostate-specific antigen, *PSAD* = PSA density. *N/A* Not Applicable, *yr* year.

*PRI-MUS positivity defined as PRI-MUS 3 with PSAD ≥ 0.15 ng/ccm and PRI-MUS 4/5.

### Diagnostic concordance between MUS and MRI

A total of 18.6% of the study population (127/682) showed a lesion on MUS. The majority of these were categorized as PRI-MUS 3 (40.9%), followed by PRI-MUS 4 (34.7%) and PRI-MUS 5 (24.4%). Due to the known diagnostic uncertainty associated with PI-RADS 3 and PRI-MUS 3 lesions, we incorporated PSAD to further stratify these findings into suspicious and non-suspicious subgroups. This led to 12.9% (88/682) of MUS cases being defined as PRIMUS positive, see Table 1.

A total of 130 patients (19.1%) underwent MRI, which revealed a PI-RADS ≥ 3 lesion in 47.7% of cases. Among these, the PI-RADS distribution was as follows: PI-RADS 3 in 30.7%, PI-RADS 4 in 53.2%, and PI-RADS 5 in 16.1%. Adjusted for PSAD, 39.2% (51/130) MRIs were defined as PI-RADS positive. The characteristics for men with an MRI are outlined in Supplemental S1.

A cross-tabulation was performed to evaluate the diagnostic agreement between PSAD-modified-PI-RADS (MRI) and PSAD-modified-PRI-MUS (MUS), see Table 2. Concordant findings were observed in 90 cases (69.2%).

**Table 2:** Overall Diagnostic Concordance between PSAD-modified-PRI-MUS and PSAD-modified PI-RADS.

		PSAD-modified-PRI-MUS		
		1, 2, 3neg	3pos, 4, 5	Total
**PSAD-modified-PI-RADS**	1, 2, 3neg	54	25	79
	3pos, 4, 5	15	36	51
	Total	69	61	130

2×2 contingency table comparing overall lesion classification (negative: 1–3 vs. positive: 3 pos./4–5) between PRI-MUS (index test) and PI-RADS (reference standard).

### Correlation of micro-ultrasound and histology

Biopsies were performed in 62 men, representing 9.1% of the total cohort (*n* = 682), detecting PCa in 29 cases, of which 18 (29.0% or 2.6% of the total cohort) were clinically significant (csPCa). The detailed subgroup characteristics are shown in Supplemental Table S2.

33 men had PSAD-modified-PRI-MUS positive lesions leading to 15 csPCa, 7 ncsPCa and 11 negative biopsies. This approach would have missed 3 csPCa and 4 ncsPCa avoiding unnecessary biopsies in 22 men or 26 men if ncsPCa is included. The PSAD-modified-PRI-MUS-based screening pathway was associated with a 6.29-fold (OR = 0.16) reduction in overdiagnosis defined as a negative biopsy and 7.22-fold (OR = 0.14) reduction in negative biopsies and ncsPCa. MUS alone without PSA, demonstrated an Odds Ratio to detect csPCa of 7.30.

When distinguishing csPCa from benign findings and non-clinically significant PCa for men with a biopsy, the PSAD-modified-PRI-MUS score demonstrated a sensitivity of 83.3% and a specificity of 59.1%, with a positive predictive value (PPV) of 45.5% and a negative predictive value (NPV) of 89.7%.

**Fig. 2:**
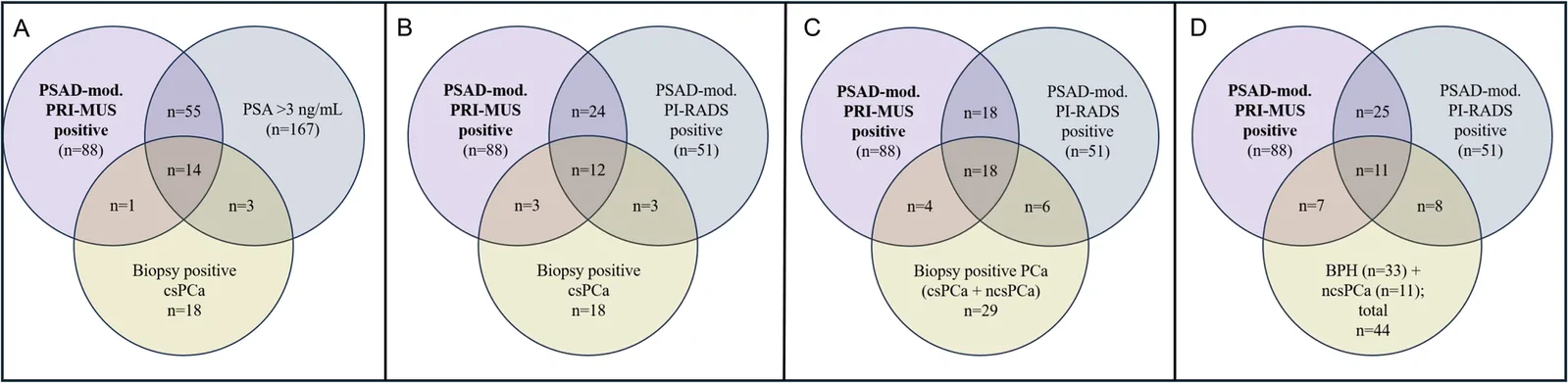
Illustration of diagnostic agreement for micro-ultrasound, PSA and MRI. Presentation of true-positive and false-positive results, along with possible overdiagnosis and underdiagnosis by micro-ultrasound (MUS) and PSA/MRI—both separately and combined. **A** Concordance between positive MUS results and PSA levels >3 ng/mL in identifying clinically significant prostate cancer (csPCa). **B** Concordance between positive MUS and positive MRI findings in identifying csPCa. **C** Concordance between positive MUS and positive MRI results in the detection of overall cancer (including both csPCa and ncsPCa). **D** Concordance between positive MUS and positive MRI results in identifying ncsPCa, alongside negative histopathology findings (non-PCa, BPH). csPCa defined as GG ≥2.

The overlap between MUS and MRI findings in the context of prostate cancer detection are shown in Figure 2. They demonstrate how each imaging modality correlates with histopathological outcomes, including csPCa, ncsPCa and benign conditions such as benign prostatic hyperplasia (BPH). A detailed analysis comparing lesion locations and cancer detection for MRI and MUS is shown in Supplemental Table S4. Of the 594 patients classified as PSAD-modified-PRI-MUS negative, 69 (11.6%) underwent prostate MRI. Overall, 54/69 (78.3%) MRIs were PSAD-modified-PI-RADS negative. Of these, 13 (24.1%) underwent biopsy (due to PSA ≥ 4; three cases in combination with PI-RADS 3) detecting one (7.7%) ncsPCa (PRI-MUS 3) and no cancer in 92.3%. In cases with positive PSAD-modified-PI-RADS findings *n* = 15 (21.7%), subsequent biopsies revealed csPCa and ncsPCa in three cases each (27.3%), while five biopsies (45.5%) were negative. A detailed biopsy analysis according to the START criteria is shown in supplemental Tables S3–S5. Targeted cores diagnosed csPCa in 42/57 (73.7%) cases compared to 51/87 (58.6%) cases by systematic biopsy on a biopsy core based analysis. An analysis of the biopsy results for both systematic and targeted biopsies compared to the lesion location is shown in supplemental Table S5.

### Biopsy outcomes based on the different screening modalities

Indications for biopsy included elevated PSA levels and/or suspicious findings on MRI and/or MUS. Table 3 presents the correlation between histopathological biopsy results and the corresponding screening parameters, including PSA, MRI, MUS, and their PSAD-modified classifications.

**Table 3:** Correlation between histopathological biopsy results and the corresponding screening.

Parameter	*N*	%	Biopsies	csPCa	csPCa/ Biopsies %	ncsPCa	no PCa
Total	682		62	18	29.0%	11	33
PSA ≥ 2 ng/mL	225	33.0%	55	17	30.9%	11	27
PSA ≥ 3 ng/mL	167	24.5%	52	17	32.7%	11	24
PSA ≥ 4 ng/mL	133	19.5%	49	16	32.7%	10	23
PSAD ≥ 0.15 ng/mL²	73	10.7%	28	13	46.4%	5	10
PRI-MUS 1 and 2	555	81.4%	23	2	8.7%	2	19
PRI-MUS ≥ 3	127	18.6%	39	16	41.0%	9	14
PRI-MUS ≥ 4	75	11.0%	30	14	46.7%	7	9
PSAD-modified- PRI-MUS positive	88	12.9%	33	15	45.5%	7	11
PSAD-modified- PRI-MUS negative	594	87.1%	29	3	10.3%	4	22
PI-RADS 1 and 2	68	52.3%	13	1	7.7%	2	10
PI-RADS ≥ 3	62	47.7%	38	15	39.5%	9	14
PI-RADS ≥ 4	43	33.1%	30	14	46.7%	9	7
No MRI	552		11	2	18.2%	0	9
PSAD-modified- PI-RADS positive	49	39.2%	34	15	44.1%	9	10
PSAD-modified- PI-RADS negative	76	60.8%	17	1	5.9%	2	14

*csPCa* clinically significant prostate cancer, *csnPCa* non-clinically significant prostate cancer.

PRI-MUS positivity defined as PRI-MUS 3 with PSAD ≥ 0.15 ng/ccm and PRI-MUS 4/5.

In comparison to a PSA based biopsy strategy with a threshold of ≥4 ng/mL, the use of PSAD-modified MUS would have reduced the number of negative biopsies by 12 (23 vs. 11), while missing only one case of csPCa (16 vs. 15) and three cases of ncsPCa (10 vs. 7).

Using the lower PSA threshold of ≥3 ng/mL, 13 negative biopsies could have been avoided (24 vs. 11), at the cost of missing two csPCa cases (17 vs. 15) and four ncsPCa cases (11 vs. 7).

In 29 PSAD-modified-PRI-MUS negative cases with biopsies performed due to elevated PSA and/or positive MRI findings, 22 (75.9%) were histologically negative, while 3 cases (10.3%) of csPCa and 4 cases (13.8%) of ncsPCa were identified.

Overall, negative MUS would have avoided 53 MRIs. Avoidable MRIs were defined as the sum of PI-RADS–positive cases with negative biopsy and PSAD-modified-PRI-MUS negative findings (*n* = 8) plus PI-RADS–negative cases with PSAD-modified-PRI-MUS negative findings (*n* = 45).

In one case, a patient with negative PSA and negative MRI underwent biopsy, solely based on a PSAD-modified-PRI-MUS positive result, which yielded no cancer.

Ten men with PI-RADS and PRI-MUS 4–5 did not undergo biopsy: five declined, two were lost to follow-up, one was monitored (age/small lesion), one had a PSA drop and the lesion was no longer detectable, and one was biopsied later (ncsPCa).

16 men with positive PRI-MUS had no MRI: 10 had normal PSA (monitored via MUS/PSA), 3 were >75 years old (active surveillance), 2 lost to follow-up, one received delayed MRI after data accrual (no lesion).

## Discussion

This retrospective pilot trial is the first to evaluate the potential of high-resolution MUS in a prostate cancer opportunistic screening cohort. While the recently published OPTIMUM trial was able to show non-inferiority of MUS compared to MRI in men at risk of prostate cancer, the role of MUS further upstream in the diagnostic pathway is unknown [5]. We sought to investigate if a MUS-based screening can be justified. Exploring further imaging modalities is needed as prostate cancer screening is on the brink of changing from the standard PSA driven approach, towards an imaging-based screening [11]. Two large screening trials incorporating an MRI screening pathway (Göteborg-2 and Stockholm-3 trials) were able to show the improved GG2 detection while reducing overdiagnosis [12, 13]. Using a PSA cut-off ≥3 ng/mL as risk threshold, 24.5% of men of our cohort would have been at risk of PCa in a screening program, which is higher than the 7–12% reported in both Göteborg-2 and Stockholm-3 trial, due to an opportunistic screening approach as is it standard of practice in Germany [12, 13]. Men received further imaging in compliance with the German prostate guideline [8]. Overall, 9.1% of men agreed to and underwent perineal systematic and targeted biopsy. Adding MUS to the risk evaluation would have reduced the number of biopsies by 26 cases (42%), missing 2 csPCa (11%). Moving MUS further upstream would have not only reduced unnecessary biopsies but also showed a high correlation with a low PSA of <2 ng/mL in MUS negative cases. A positive MRI detected only 3 csPCa while 5 biopsies remained negative in MUS negative cases. In a prospective cohort of 425 men with elevated PSA levels Beatrici et al. also showed that a negative MUS was able to reduce overdiagnosis and reduce the number of MRIs performed, while missing only 2 csPCa [14]. Further, MRI could have been avoided in 53 cases, and the rate of MUS and MRI concordance was 69.2%. This shows the potential of MUS as a first line tool for screening in addition to the PSA staged imaging pathway.

The value of MRI and conventional ultrasound was compared to a PSA based screening in the PROSTAGRAM trial. While MRI doubled the number of men detected with csPCa when compared to a PSA ≥ 3 ng/mL, ultrasound did not show a benefit to PSA alone [15]. To reduce overdiagnosis, the authors recommended PI-RADS 4/5 as a cut-off; a notion also supported in a recent review by Schoots et al. [16]. Adopting a PRI-MUS 4/5 as cut-off would have led to 4 missed csPCa in our analysis, while avoiding 3 biopsies compared to the chosen threshold.

To reduce the impact of equivocal PRI-MUS 3 lesions, which corresponds to PI-RADS 3 often describing inflammatory changes, we propose a novel PSA density-based PRI-MUS 3 classification which was able to avoid unnecessary biopsies. Overall, the low rate of PRI-MUS 3 in our cohort was comparable to the rate of PI-RADS 3 (6% resp. 7%) in MRI as first-line screening trials (IP1-PROSTAGRAM resp. VISIONING) and the 10% in the Göteborg-2 trial [12, 15, 17].

The overdiagnostic aspect of equivocal lesions was further reduced to 1.9% by the introduction of the PSAD-based PRI-MUS 3 assessment.

The OPT screening trial, with a PI-RADS 3 rate of 20% due to the inclusion of younger men, was able to show that incorporating PSAD for PI-RADS 3 lesions improved csPCa detection [13, 14]. Based on the retrospective data from the STHLM3-MRI trial which showed no benefit in using PSAD for PI-RADS 3 cases, the authors of a recently published review on MRI based screening recommend using PSAD based biopsy decisions with caution [11, 15]. Further studies will have to evaluate the optimal PI-RADS/PRI-MUS lesion cut-off for the imaging-based screening pathway.

The introduction of first line MRI in the screening has also raised the question of the optimal PSA cut-off if incorporated as a PSA staged screening test. In the Göteborg-2 trial, 4.6% of men with PSA levels between 1.8 and 3 ng/mL were diagnosed with GG2 cancer due to a positive MRI, without an increase in GG1 cancers [18]. The impact of a delayed cancer detection at a higher PSA threshold in this subgroup of men remains uncertain. A similar effect can be postulated for a MUS-based screening.

Overall, the diagnostic performance of PSAD-modified-PRI-MUS seems to align with the MRI screening data, particularly in excluding clinically insignificant cases [13, 18]. These findings support its potential use of MUS as a triage or alternative screening tool when MRI is inconclusive, unavailable, or contraindicated.

As the PRI-MUS scoring system was developed for men at risk of prostate cancer, future studies will also have to evaluate the optimal scoring in a screening setting, especially if younger men are included. PRI-MUS assessment in younger men may face similar difficulties as in the PROBASE trial, with a high number of equivocal MRI findings, overall low cancer incidence and high dependency of reader experience potentially due to the higher overall density of the peripheral zone in younger men [19].

This study is not without limitations. The analysis was performed retrospectively at a high- volume MUS center with two experienced users in both MRI/MUS fusion biopsy and MUS usage as a TRUS replacement, limiting generalizability. The study did not follow a randomized design, and not all patients underwent multiparametric MRI or prostate biopsy, as these procedures were only performed when clinically indicated. This trial was not focused on the detailed biopsy results comparing MRI to MUS, which presents a different patient cohort; therefore, the number of patients biopsied was low. Consequently, the study cohort reflects everyday clinical practice rather than a pre-selected clinical trial population. Biopsy decisions were based on all clinically available parameters with a possible bias towards the stand-alone MUS performance and not all men followed the biopsy recommendation. Further standardization of MUS scanning and PRI-MUS interpretation will be necessary for a broad integration into the PCa screening pathway. Therefore, in a prospective trial setting, the MUS-based biopsy indication may lead to a different benefit to harm ratio. Further, all cases were assessed prior to the publication of the prospective OPTIMUM trial which showed non-inferiority of MUS vs. MRI which may have influenced the decision-making process in PRI-MUS positive but PSA/MRI negative cases [5]. All men included opted for opportunistic screening and the impact of MUS in a prospective screening setting warrants further evaluation. MRI was not available or performed for all men with a known PSA but stable elevation, reducing the number of men with both PSA and MRI as a reference test to MUS. Nonetheless this trial also represents the use of MUS in real-life setting.

This retrospective pilot trial is the first to provide insight into the potential of a MUS-based prostate cancer screening. Both first line use before or instead PSA or as part of a PSA staged assessment seem feasible and warrant a prospective trial to evaluate MUS compared to PSA and MRI base screening approaches.

## Supplementary information

Supplemental Tables

## Data Availability

The datasets generated during and/or analysed during the current study are available from the corresponding author on reasonable request.
