# Supplementary material for: The value of micro-ultrasound for prostate cancer screening: A retrospective real-world feasibility study
Source: Prostate Cancer Prostatic Dis. 2026 Jan 26;29(4):799–805. doi: 10.1038/s41391-026-01075-x (PMC13623632; doi:10.1038/s41391-026-01075-x)
Supplement: Supplementary file 1 [file 41391_2026_1075_MOESM1_ESM.doc]

**Supplement**

Table S1. Characteristics MRI

| **Characteristic** | **All** | **MRI done** | **MRI not done** | **p value** |
| --- | --- | --- | --- | --- |
| Number of patients (%) | 682 (100) | 130 (19.1) | 552 (80.9) | N/A |
| Age [yr], median (IQR) | 59 (51.0 – 66.0) | 64 (59.8 – 72.3) | 57 (50.0 – 64.0) | N/A |
| positive family history of PCa (%) | 65 (9.5) | 10 (7.7) | 55 (10.0) | N/A |
| Suspicious DRE (%) | 28 (4.1) | 16 (12.3) | 12 (2.2) | N/A |
| Median prostate volume [mL], (IQR) | 26 (21 – 40) | 45 (32.0 - 63.5) | 25 (20.0 - 35.0) | N/A |
| Median PSA [ng/mL], (IQR) | 1.2 (0.6 – 3.6) | 5.9 (3.88 - 8.65) | 0.9 (0.5 - 1.9) | <0.001 |
| Median PSAD [ng/mL²], (IQR) | 0.04 (0.03 – 0.09) | 0.12 (0.07 - 0.19) | 0.03 (0.02 - 0.06) | <0.001 |
| PSA ≥4 ng/mL (%) | 133 (19.5) | 90 (69.2) | 43 (7.8) | N/A |
| PSA ≥3 ng/mL (%) | 167 (24.5) | 104 (80.0) | 63 (11.4) | N/A |
| PSA ≥2 ng/mL (%) | 225 (33.0) | 115 (88.5) | 110 (19.9) | N/A |
| PSAD ≥0.15 ng/mL² (%) | 73 (10.7) | 48 (36.9) | 25 (4.5) | <0.001 |
| PRI-MUS 1/2 (%) | 555 (81.4) | 59 (45.4) | 496 (89.9) | N/A |
| PRI-MUS 3 (%) | 52 (7.6) | 18 (13.8) | 34 (6.2) | N/A |
| PRI-MUS 4 (%) | 44 (6.5) | 31 (23.8) | 13 (2.4) | N/A |
| PRI-MUS 5 (%) | 31 (4.5) | 22 (16.9) | 9 (1.6) | N/A |
| Abbreviations: DRE = digital rectal exam; IQR = interquartile range; MUS = Micro-ultrasound; MRI = multiparametric magnet resonance imaging; PCa = prostate cancer; PI-RADS = Prostate Imaging Reporting and Data System; PRI-MUS = Prostate Risk Identification using Micro-ultrasound; PSA = prostate-specific antigen; PSAD = PSA density. N/A = Not Applicable; yr = year. | | | | |

Table S2. Characteristics Biopsy

| **Characteristic** | **All** | **Biopsy** | **No Biopsy** | **p value** |
| --- | --- | --- | --- | --- |
| Number of patients (%) | 682 (100) | 62 (9.1) | 620 (90.9) | N/A |
| Age [yr], median (IQR) | 59 (51.0 – 66.0) | 66.0 (60.0 - 75.0) | 58 (51.0 - 65.0) | N/A |
| positive family history of PCa (%) | 65 (9.5) | 3 (4.8) | 62 (10.0) | N/A |
| Suspicious DRE (%) | 28 (4.1) | 14 (22.6) | 14 (2.3) | N/A |
| Median prostate volume [mL], (IQR) | 26 (21 – 40) | 46.0 (31.75 - 62.0) | 25.0 (20.0 - 38.0) | N/A |
| Median PSA [ng/mL], (IQR) | 1.2 (0.6 – 3.6) | 6.8 (4.8 - 10.0) | 1.1 (0.6 - 2.5) | <0.001 |
| Median PSAD [ng/mL²], (IQR) | 0.04 (0.03 – 0.09) | 0.15 (0.06 - 0.30) | 0.02 (0.00 - 0.04) | <0.001 |
| PSA ≥4 ng/mL (%) | 133 (19.5) | 49 (77.4) | 84 (13.5) | N/A |
| PSA ≥3 ng/mL (%) | 167 (24.5) | 52 (83.9) | 115 (18.5) | N/A |
| PSA ≥2 ng/mL (%) | 225 (33.0) | 55 (88.7) | 170 (27.4) | N/A |
| PSAD ≥0.15 ng/mL² (%) | 73 (10.7) | 28 (45.2) | 45 (7.3) | <0.001 |
| PRI-MUS 1/2 (%) | 555 (81.4) | 23 (37.1) | 532 (85.8) | <0.001 |
| PRI-MUS 3 (%) | 52 (7.6) | 9 (14.5) | 43 (6.9) | 0.032 |
| PRI-MUS 4 (%) | 44 (6.5) | 10 (16.1) | 34 (5.5) | 0.001 |
| PRI-MUS 5 (%) | 31 (4.5) | 20 (32.3) | 11 (1.8) | <0.001 |
| MRI (%) | 130 (19.1) | 51 (82.3) | 79 (12.7) | <0.001 |
| PI-RADS 1/2 (%) | 68 (52.3) | 13 (25.5) | 55 (69.6) | N/A |
| PI-RADS 3 (%) | 19 (14.6) | 8 (15.7) | 11 (13.9) | N/A |
| PI-RADS 4 (%) | 33 (25.4) | 23 (45.1) | 10 (12.7) | N/A |
| PI-RADS 5 (%) | 10 (7.7) | 7 (13.7) | 3 (3.8) | N/A |
| Abbreviations: DRE = digital rectal exam; IQR = interquartile range; MUS = Micro-ultrasound; MRI = multiparametric magnet resonance imaging; PCa = prostate cancer; PI-RADS = Prostate Imaging Reporting and Data System; PRI-MUS = Prostate Risk Identification using Micro-ultrasound; PSA = prostate-specific antigen; PSAD = PSA density. N/A = Not Applicable; yr = year. | | | | |

*Table S3. Characteristics of systematic and MRI targeted biopsy*

|  | **Systematic biopsy** | **Targeted biopsy** |
| --- | --- | --- |
| Number of biopsy cores | 671 | 231 |
| Number of positive cores | 87 | 57 |
| Positive rate (%) | 13.0 | 24.7 |
| Rate of clinically significant cancer cores | 51/87 (58.6) | 42/57 (73.7) |
| Median cancer core length [mm], IQR | 2.0 (1.0-3.8) | 5.0 (2.4-10.0) |
| Median cancer core percentage [%], IQR | 16.0 (7.0-27.0) | 34.5 (16.5-73.5) |
| Grade Group |  |  |
| 1 | 12 cases / 36 cores | 5 cases / 15 cores |
| 2 | 6 cases / 17 cores | 8 cases / 21 cores |
| 3 | 3 cases / 11 cores | 5 cases / 12 cores |
| 4 | 6 cases / 25 cores | 4 cases / 6 cores |
| 5 | 0 cases / 0 cores | 1 case / 3 cores |
| Abbreviations: IQR = interquartile range; MRI = multiparametric magnet resonance imaging | | |

Table S4. Lesion Location and Cancer Detection for both MRI and Micro-Ultrasound

|  | **Concordance** | | |
| --- | --- | --- | --- |
| **Localization** | **MUS/MRI** | **MRI/PCa** | **MUS/PCa** |
| **Ventral medial** | 68.8% (11/16) | 52.2% (12/23) | 45.5% (10/22) |
| **Ventral lateral** | 58.3% (7/12) | 45.0% (9/20) | 33.3% (7/21) |
| **Mid gland lateral** | 50.0% (10/20) | 50.0% (12/24) | 36.4% (8/22) |
| **Transition zone** | 9.1% (1/11) | 17.4% (4/23) | 8.0% (2/25) |
| **Apex** | 33.3% (6/18) | 17.9% (5/28) | 29.6% (8/27) |
| **Base** | 41.2% (7/17) | 16.7% (3/18) | 52.6% (10/19) |
| **Total** | 44.7% (42/94) | 33.1% (45/136) | 33.1% (45/136) |
| Abbreviations: MUS = Micro-ultrasound; MRI = multiparametric magnet resonance imaging; PCa = prostate cancer | | | |

*Table S5. Cancer detection for patient and lesion-based analysis in regard to lesion location and biopsy approach*

|  | **Cancer diagnosis** | | | | **Cancer cores** | | | |
| --- | --- | --- | --- | --- | --- | --- | --- | --- |
|  | **Standard biopsy** | | **Targeted biopsy** | | **Standard biopsy** | | **Targeted biopsy** | |
|  | **ncsPCa** | **csPCa** | **ncsPCa** | **csPCa** | **ncsPCa** | **csPCa** | **ncsPCa** | **csPCa** |
| **Ventral medial** | 8 | 4 | 2 | 6 | 8 | 4 | 2 | 11 |
| **Ventral lateral** | 6 | 5 | 5 | 3 | 7 | 8 | 5 | 4 |
| **Mid gland lateral** | 4 | 6 | 4 | 5 | 5 | 7 | 6 | 7 |
| **Transition zone** | 9 | 8 | 1 | 3 | 10 | 8 | 2 | 3 |
| **Apex** | 2 | 6 | 2 | 8 | 2 | 6 | 2 | 13 |
| **Base** | 4 | 4 | 1 | 5 | 4 | 6 | 1 | 5 |
| **Total** | 33 | 33 | 15 | 30 | 36 | 39 | 18 | 43 |

Abbreviations: csPCa = clinically significant prostate cancer; IQR = interquartile range; MRI = multiparametric magnet resonance imaging; ncsPCa = non-clinically significant prostate cancer
